# Evaluation of Knowledge, Attitude, and Practices of Compulsory Rotating Medical Interns in Managing Obstetric Emergencies in a Tertiary Hospital

**DOI:** 10.7759/cureus.99578

**Published:** 2025-12-18

**Authors:** Karuvizhi P Mahalekshmi, Reshmi S, Meena T S, Sornam MS

**Affiliations:** 1 Obstetrics and Gynaecology, Sree Balaji Medical College and Hospital, Chennai, IND

**Keywords:** intern doctors, maternal-fetal health, obstetric emergencies, postpartum haemorhage, simulation training

## Abstract

Background

Obstetric emergencies such as postpartum haemorrhage (PPH), eclampsia, and shoulder dystocia remain major causes of maternal mortality in low- and middle-income countries. The preparedness of frontline providers, including Compulsory Rotatory Medical Interns (CRMIs), is critical in preventing avoidable maternal deaths. Simulation-based obstetric emergency training has demonstrated global success in improving clinical competence; however, structured evaluation of interns’ readiness in Indian tertiary settings remains limited. This study was conducted to assess the knowledge, attitude, and practices (KAP) of CRMIs regarding obstetric emergency management and to identify factors influencing preparedness.

Methods

A hospital-based cross-sectional study was conducted among 100 CRMIs in a tertiary care teaching institution. Data were collected using a validated semi-structured questionnaire assessing demographic characteristics, knowledge of key obstetric emergencies, attitude toward team-based care, and self-reported clinical practices. Statistical analysis included descriptive proportions and association testing using chi-square and odds ratios with 95 % confidence intervals. Ethical clearance and informed consent were obtained.

Results

The mean age of participants was 24.1 ± 1.2 years, and 54 interns (54%) were females. A total of 70 interns (70%) were currently posted in obstetrics, while 83 (83%) had previously completed an obstetrics and gynaecology (OBG) posting. Formal training in obstetric emergencies had been undertaken by 79 interns (79%). Overall, 84 interns (84%) demonstrated adequate knowledge, 86 (86%) showed a positive attitude, and 74 (74%) exhibited good practice in managing obstetric emergencies. Knowledge adequacy showed significant associations with current OBG posting (47/70; p = 0.047), prior posting (75/83; p = 0.043), formal training (67/79; p = 0.021), and higher exposure to obstetric emergencies (approx. 56/70; p = 0.035).

Conclusion

The study demonstrates encouraging preparedness among CRMIs for managing obstetric emergencies, particularly among those with structured training and repeated exposure. Integrating mandatory simulation-based obstetric emergency programmes such as Advanced Life Support in Obstetrics (ALSO) or Basic Life Support in Obstetrics (BLSO) before CRMI posting is strongly recommended to bridge the gap between theoretical knowledge and hands-on clinical proficiency. Institutional adoption of periodic drills and refresher training will further strengthen emergency readiness and support national maternal-health goals.

## Introduction

Maternal and newborn health remains one of the most critical indicators of the strength and responsiveness of any healthcare system. Despite remarkable progress achieved through global public health initiatives, maternal morbidity and mortality continue to pose significant challenges, particularly in low- and middle-income countries where preventable complications contribute disproportionately to maternal deaths. According to the World Health Organization (WHO), approximately 295,000 women die each year due to pregnancy- and childbirth-related causes, the vast majority of which could be avoided through timely recognition and skilled management of obstetric emergencies [1]. To address this burden, the WHO manual Managing Complications in Pregnancy and Childbirth provides a comprehensive, step-by-step guide to handling life-threatening situations, including postpartum haemorrhage (PPH), eclampsia, sepsis, and obstructed labour. This manual has formed the backbone of structured emergency obstetric training initiatives globally and is widely adopted across many countries, including India [1].

In parallel, several countries have implemented competency-based training models to standardize emergency obstetric care. The American Academy of Family Physicians developed the Advanced Life Support in Obstetrics (ALSO) course to strengthen clinicians’ ability to manage critical events in labour and delivery through evidence-based algorithms and hands-on simulation [2]. Widely implemented across North America, Australia, and parts of Asia, the ALSO programme integrates cognitive learning with practical skills and encourages rapid, team-based responses to emergencies such as maternal collapse, shoulder dystocia, cord prolapse, and severe PPH. Similarly, in the United Kingdom, the Royal College of Obstetricians and Gynaecologists (RCOG) introduced the Emergency Obstetric Drill training toolkit that emphasizes scenario-based simulation, leadership, team communication, and coordinated decision-making under pressure [3]. Variants of this model have been replicated in Europe, the Middle East, and South Asia.

Despite the presence of such structured training modules, global evidence highlights variability in knowledge transfer, retention, and translation into clinical practice. A systematic review conducted across the UK, Tanzania, and Nigeria found that emergency obstetric care training significantly improved participants’ knowledge and procedural competence; however, long-term retention and application in real-world settings remained inconsistent, underscoring the need for continuous reinforcement and refresher training [4]. Similarly, an on-site obstetric emergency training programme in Zimbabwe demonstrated notable improvements in response times and clinical decision-making among doctors, nurses, and midwives following hands-on simulation drills [5]. The Zimbabwean experience emphasized that sustained improvement required ongoing mentorship, supportive hospital policies, and regular audit mechanisms.

In high-income countries, such as Australia, completion of the ALSO programme led to substantial increases in clinicians’ confidence and perceived competence in managing conditions including eclampsia and shoulder dystocia, with the multidisciplinary approach identified as a key contributor to improved readiness [6]. Collectively, these international experiences underscore that continuous, simulation-supported training can bridge the gap between knowledge and practice.

Beyond clinical training, knowledge, attitude, and practice (KAP) assessments have long been utilized to evaluate healthcare workers’ competencies, perceptions, and behavioural patterns. Although widely applied in communicable disease research, KAP frameworks are equally useful in assessing emergency preparedness. For example, a KAP study among pregnant women in Karachi during the COVID-19 pandemic revealed significant deficiencies in awareness and preventive practices, demonstrating how structured assessments help identify educational needs and tailor interventions [7]. Another hospital-based study in the United States assessing KAP among obstetric nurses caring for HIV-positive women found that better attitudes and practices correlated strongly with prior training and institutional support [8]. Similar findings in developing countries reinforce that knowledge alone is insufficient; positive attitudes and consistent clinical behaviour are equally essential.

In India, although maternal mortality has declined steadily, it remains higher than global benchmarks. Most maternal deaths in tertiary settings arise from delays in recognizing emergencies or inadequate initial management. Compulsory Rotatory Medical Interns (CRMIs) frequently serve as the first point of contact during obstetric emergencies, especially in labour wards and night-duty settings. Their preparedness, competence, and confidence critically influence the trajectory of maternal outcomes. Evidence from a cross-sectional study in New Delhi during the COVID-19 pandemic showed that prior exposure and educational level significantly influenced health-seeking behaviour among pregnant women, highlighting the broader impact of structured training on healthcare delivery [9].

The COVID-19 pandemic also exposed major gaps in emergency readiness. An online global survey of maternal and newborn health professionals from Africa, Asia, and Europe revealed severe constraints due to workforce shortages, supply disruptions, and psychological stress, all of which adversely affected emergency preparedness [10]. These challenges strengthen the argument for institutionalized, sustainable training frameworks.

Several competency-based modules have demonstrated success in resource-limited settings. The Helping Mothers Survive: Bleeding After Birth programme implemented in Tanzania, Uganda, and India significantly improved PPH management skills among healthcare workers, with regular reinforcement being essential for sustained competence [11]. Similarly, studies on neonatal resuscitation practices in Mozambique identified cognitive errors, delayed decision-making, and poor situational awareness as major contributors to adverse outcomes, recommending greater emphasis on anticipation, prioritization, and communication skills in training curricula [12]. Against this global and regional backdrop, there is a critical need to evaluate the preparedness of CRMIs in Indian tertiary hospitals. Understanding their knowledge, attitudes, and practices will enable targeted, simulation-based training interventions that align with national and global maternal health priorities. This study aimed to evaluate the level of knowledge, attitude, and clinical practices of CRMIs regarding common obstetric emergencies such as postpartum hemorrhage, hypertensive disorders, shoulder dystocia, obstructed labour and maternal sepsis using a structured and validated questionnaire and to identify associations between socio-demographic factors, clinical exposure, and KAP scores among CRMIs, and determine the key barriers and enablers influencing their preparedness in managing obstetric emergencies.

## Materials and methods

Study design and setting

This study employed a hospital-based, cross-sectional descriptive design to evaluate the KAP of CRMIs in the identification and management of obstetric emergencies. The research was conducted in the department of obstetrics and gynaecology (OBG) at Sree Balaji Medical College and Hospital (SBMCH), Chennai, a tertiary care teaching institution that manages a large volume of routine and high-risk pregnancies. The department’s clinical workload and diverse case mix provided an appropriate environment for assessing interns’ preparedness for obstetric emergencies. Data collection took place over a three-month period, from October 2025 to December 2025, allowing adequate time for recruitment, questionnaire administration, and completion of data collection activities.

Study population

The study population consisted of all CRMIs who were posted in the departments of OBG and emergency medicine during the study period. As these interns often formed the first line of response during obstetric emergencies, especially during night duties and initial patient assessments, their inclusion was essential for understanding real-world readiness. Only those actively involved in their postings at the time of data collection were considered eligible.

Inclusion and exclusion criteria

CRMIs were included if they were posted in the OBG or emergency medicine departments, had completed a minimum of two weeks in their respective postings, and had provided written informed consent. This ensured adequate clinical exposure to obstetric care routines and emergency scenarios.

Interns were excluded if they had already undergone formal obstetric emergency training programmes such as ALSO or Basic Life Support in Obstetrics (BLSO), as prior structured training might have influenced KAP scores. CRMIs who were on leave, unavailable during data collection, or unwilling to participate were also excluded from the study.

Study period

The study was carried out from October 2025 to December 2025 at SBMCH, Chennai, during which all data collection and preliminary analysis activities were completed.

Sample size calculation

The sample size was calculated using a single-proportion formula, referencing a study by Naz et al. [7], which reported that 75% of participants demonstrated adequate knowledge of infection control practices. Using this proportion (p = 0.75), a 95% confidence interval (CI) (Z = 1.96), and a 9% margin of error (d = 0.09), the initial sample size was obtained. After adjusting for a 10% non-response rate, the final sample requirement was calculated to be approximately 100. The final sample size was therefore rounded to 100 CRMIs, ensuring adequate power to assess associations between variables.

Ethical considerations

Ethical approval for the study was obtained from the Institutional Human Ethics Committee (IHEC) of SBMCH (002/SBMCH/IHEC/2025/2537) before commencement. Written informed consent was obtained from each participant after explaining the study objectives, procedures, and measures taken to ensure confidentiality. No identifying information was collected, and all responses were anonymized during data handling. Participation was voluntary, and interns were informed that refusal or withdrawal would not affect their academic evaluations or internship responsibilities.

Data collection tools and process

Data were collected using a structured, pre-validated, self-administered questionnaire designed specifically for this study. The questionnaire used in this study was developed by the authors (Karuvizhi PM, TS Meena, and Reshmi) specifically for the purpose of evaluating the KAP of CRMIs in managing obstetric emergencies. The tool was not taken from any previously published questionnaire, nor was any copyrighted instrument reproduced. However, in order to ensure strong content validity and alignment with globally established competencies, the authors drew conceptual guidance from internationally recognized open-access training resources, the WHO Emergency Obstetric Care Training Manual (open access) and the Managing Complications in Pregnancy and Childbirth (MCPC) manual [13,14]. Additional ideas for item framing were adapted conceptually, not reproduced verbatim, from validated KAP studies conducted by Walker et al., Farley et al., and Naz et al. [6-8]. These studies were used only as conceptual references to guide domain selection and structure; no copyrighted survey items were copied.

The questionnaire comprised four sections attached in the appendix (Table 6). Section A captured sociodemographic data such as age, gender, medical college, duration of internship, previous OBG postings, and prior exposure to emergency drills. Section B assessed knowledge using 15 multiple-choice questions covering recognition and immediate management of common obstetric emergencies, including PPH, eclampsia, shoulder dystocia, sepsis, and obstructed labour. These items reflected WHO EmOC competencies and ALSO curriculum priorities. Section C contained 10 Likert-scale statements evaluating attitudes towards emergency preparedness, confidence levels, teamwork, communication, and perceived challenges. These items were adapted from validated attitude measurement tools used in obstetric KAP research. Section D assessed practice through self-reported involvement in actual emergency scenarios, including participation in PPH and eclampsia management, use of emergency medications, experience with labour monitoring, and familiarity with escalation protocols.

Validation and pilot testing

The questionnaire underwent content validation by three experts from OBG and medical education, who evaluated the relevance, clarity, and appropriateness of all items. After incorporating expert inputs, the tool was pilot-tested among 10 CRMIs who met the eligibility criteria but were excluded from the main study. The pilot test assessed clarity, ease of administration, and average response time. Based on feedback, minor adjustments were made to improve item clarity. Internal consistency of the attitude and practice sections was assessed using Cronbach’s alpha, with the reliability coefficient achieving the target value of ≥0.7.

Scoring system

Knowledge scores were calculated by awarding one point for each correct answer, for a maximum total of 15. Scores of 11 or higher were categorized as indicating adequate knowledge. Attitude responses were scored on a five-point Likert scale, with reverse scoring applied to negatively phrased items; higher overall scores reflected more positive attitudes towards managing obstetric emergencies. Practice responses were scored using binary or Likert-type responses, with aggregated values forming the overall practice score. Cutoff points for adequate and inadequate practice were defined prior to analysis based on distribution patterns from the pilot study.

Statistical analysis

Data were entered into MS Excel (Microsoft Corporation, Redmond, Washington, United States) and subsequently analyzed using IBM SPSS Statistics for Windows, Version 26 (Released 2018; IBM Corp., Armonk, New York, United States). Descriptive statistics, including mean, standard deviation, frequency, and percentage, were calculated to summarize demographic characteristics and KAP scores. Associations between demographic variables and categorized KAP scores were tested using logistic regression tests, where appropriate, and using an odds ratio. A p-value less than 0.05 was considered statistically significant.

## Results

The study included 100 CRMIs with a mean age of 24.1 ± 1.2 years. Of the participants, 54 (54%) were females, and 46 (46%) were males. At the time of the survey, 70 interns (70%) were posted in the OBG department, while 30 (30%) were posted in other departments. A majority, 83 interns (83%), had previously completed an OBG posting, and 79 (79%) reported having received formal obstetric emergency training such as ALSO or BLSO, whereas 21 (21%) had not undergone any structured training. Regarding clinical exposure, 42 interns (42%) had attended more than 10 normal deliveries, 26 (26%) had attended 6-10 deliveries, 20 (20%) had attended 1-5, and 12 (12%) had not attended any normal delivery. Exposure to obstetric emergencies also varied: 35 interns (35%) had witnessed more than 10 emergencies, another 35 (35%) had witnessed 6-10, 22 (22%) had observed 1-5 cases, and 8 (8%) had not witnessed any obstetric emergency (Table 1).

**Table 1:** Demographic distribution among the study participants (n = 100) OBG: obstetrics and gynaecology; ALSO:  Advanced Life Support in Obstetrics; BLSO: Basic Life Support in Obstetrics

Category	Frequency (n) (n = 100)	Percentage (%)
Age (in years)		
(Mean ± SD)	24.1 ± 1.2	–
Gender		
Male	46	46.0
Female	54	54.0
Currently posted in OBG		
Yes	70	70.0
No	30	30.0
Previously completed an OBG posting		
Yes	83	83.0
No	17	17.0
Formal training in obstetric emergencies (ALSO/BLSO, etc.)		
Yes	79	79.0
No	21	21.0
Number of normal deliveries attended		
0	12	12.0
1-5	20	20.0
6-10	26	26.0
>10	42	42.0
Number of obstetric emergencies witnessed		
0	8	8.0
1-5	22	22.0
6-10	35	35.0
>10	35	35.0

Table 2 shows that most CRMIs demonstrated a strong theoretical understanding of obstetric emergencies. A total of 91 interns (91%) correctly identified oxytocin as the first-line drug for PPH, while 89 interns (89%) recognized haemorrhage as the leading cause of maternal death. Knowledge regarding the drug of choice for eclampsia was also high, with 87 interns (87%) identifying magnesium sulfate correctly. Similarly, 80 interns (80%) were able to recognize maternal sepsis using a pulse rate greater than 100 per minute. Slightly lower, but still satisfactory, proportions were noted for items assessing partograph use and active management of the third stage of labour (AMTSL) components, with 79 interns (79%) and 73 interns (73%) answering these correctly, respectively.

**Table 2:** Distribution of knowledge among the study participants (n = 100) PPH: postpartum haemorrhage; AMTSL: active management of the third stage of labour

Knowledge item (n = 100)	Correct response n (%)
First-line drug for PPH (oxytocin)	91 (91.0)
Drug for eclampsia (magnesium sulfate)	87 (87.0)
Use of partograph (monitoring labor progress)	79 (79.0)
Initial step for shoulder dystocia (McRoberts)	74 (74.0)
Red flag for sepsis (pulse >100/min)	80 (80.0)
AMTSL components	73 (73.0)
Uterine atony is best assessed by tone	79 (79.0)
Recommended magnesium sulfate loading dose (IV)	77 (77.0)
“Golden hour” definition (1 hour)	72 (72.0)
Most common cause of maternal death in India (haemorrhage)	89 (89.0)

Table 3 shows that the majority of CRMIs demonstrated a positive and proactive attitude towards managing obstetric emergencies. A total of 81 interns (81%) reported feeling confident in managing PPH, while 80 interns (80%) expressed confidence in administering emergency medications. Furthermore, 85 interns (85%) agreed that simulation-based drills should be mandatory, highlighting their strong preference for hands-on, practice-oriented learning. Confidence in using a partograph was also notable, with 79 interns (79%) indicating they felt capable of using it effectively. Similarly, 85 interns (85%) believed they communicated well with team members during critical situations. Although a smaller group, approximately 15 interns (15%), admitted to hesitation without supervision or fear of making mistakes, these responses were relatively few and suggest an overall trend toward increasing self-assurance among the interns.

**Table 3:** Distribution of attitude responses among the study participants (n = 100)

Attitude statement	Strongly disagree n (%)	Disagree n (%)	Neutral n (%)	Agree n (%)	Strongly agree n (%)
1. I feel confident managing postpartum haemorrhage	3 (3.0)	6 (6.0)	10 (10.0)	45 (45.0)	36 (36.0)
2. My UG training prepared me well for obstetric emergencies	5 (5.0)	8 (8.0)	12 (12.0)	44 (44.0)	31 (31.0)
3. I hesitate without supervision in obstetric emergencies	20 (20.0)	30 (30.0)	15 (15.0)	25 (25.0)	10 (10.0)
4. I am confident administering emergency medications	2 (2.0)	6 (6.0)	12 (12.0)	46 (46.0)	34 (34.0)
5. Simulation-based drills should be mandatory	0 (0.0)	0 (0.0)	5 (5.0)	35 (35.0)	60 (60.0)
6. I fear making mistakes in emergencies	18 (18.0)	35 (35.0)	12 (12.0)	20 (20.0)	15 (15.0)
7. I feel confident using a partograph	3 (3.0)	8 (8.0)	10 (10.0)	50 (50.0)	29 (29.0)
8. I communicate well with team in critical scenarios	1 (1.0)	4 (4.0)	10 (10.0)	50 (50.0)	35 (35.0)
9. I can take responsibility when needed	1 (1.0)	3 (3.0)	6 (6.0)	50 (50.0)	40 (40.0)
10. Structured training would improve my performance	0 (0.0)	1 (1.0)	4 (4.0)	40 (40.0)	55 (55.0)

Table 4 indicates that a considerable proportion of CRMIs demonstrated strong practical exposure to managing obstetric emergencies. A total of 50 interns (50%) reported having independently handled at least one obstetric emergency, while an even larger majority, 76 interns (76%), had administered essential emergency medications such as oxytocin or magnesium sulfate, reflecting adequate hands-on skill application in critical scenarios. Nearly two-thirds, 68 interns (68%), stated that they had used a partograph to monitor labour, and 81 interns (81%) reported that they “always” or “often” escalated high-risk cases to senior clinicians, demonstrating responsible and collaborative clinical behaviour. In addition, 72 interns (72%) regularly monitored vital signs in labour patients, and 58 interns (58%) had participated in simulation-based drill sessions, indicating a good level of engagement in practical skills training. Although most interns, 69 (69%), frequently referred to emergency protocols, a smaller proportion reported doing so only occasionally, highlighting an area where adherence could be further strengthened.

**Table 4:** Distribution of practise responses among the study participants (n = 100)

Practice	n (%)
Have you handled an obstetric emergency independently?	
Yes	50 (50.0)
No	50 (50.0)
Have you administered any obstetric emergency drug (e.g., oxytocin, magnesium sulfate)?	
Yes	76 (76.0)
No	24 (24.0)
Have you used a partograph to monitor labor?	
Yes	68 (68.0)
No	32 (32.0)
How often do you escalate high-risk labor to a senior?	
Always	45 (45.0)
Often	36 (36.0)
Sometimes	12 (12.0)
Rarely	5 (5.0)
Never	2 (2.0)
Do you regularly monitor vitals in labor patients?	
Always	40 (40.0)
Often	32 (32.0)
Sometimes	18 (18.0)
Rarely	8 (8.0)
Never	2 (2.0)
Have you participated in a simulation/drill for obstetric emergencies?	
Yes	58 (58.0)
No	42 (42.0)
How often do you refer to obstetric emergency protocols?	
Always	38 (38.0)
Often	31 (31.0)
Sometimes	18 (18.0)
Rarely	8 (8.0)
Never	5 (5.0)

**Figure 1:**
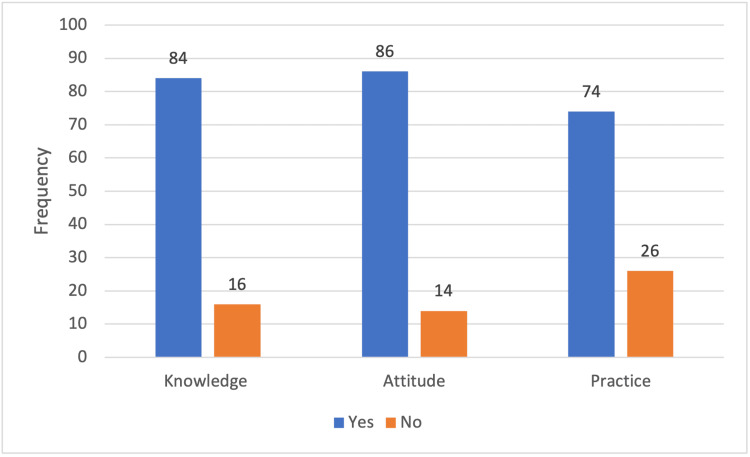
Distribution of knowledge, attitude, and practice levels among study participants in managing obstetric emergencies

Overall, 84 interns (84%) demonstrated adequate knowledge, whereas 16 interns (16%) had inadequate knowledge, indicating a largely well-informed group with some areas requiring further improvement. Similarly, 86 interns (86%) displayed a positive attitude towards managing obstetric emergencies, while 14 interns (14%) showed neutral or negative attitudes. With respect to practical skills, 74 interns (74%) demonstrated good practice, whereas 26 interns (26%) showed poor practice. Taken together, these findings highlight strong foundational preparedness among CRMIs, while also underscoring the need for continued reinforcement through targeted training interventions (Figure 1).

Table 5 presents the relationship between various demographic and experiential factors and the level of knowledge among CRMIs. The findings indicate that being currently posted in OBG, previously completing an OBG posting, and having formal training in obstetric emergencies were all significantly associated with higher knowledge scores. Interns currently posted in OBG were less likely to have inadequate knowledge compared to those not posted (p = 0.047). Similarly, those who had previously completed an OBG posting (p = 0.043) and those with formal obstetric emergency training (p = 0.021) demonstrated significantly better knowledge levels. Clinical exposure also showed a strong influence on knowledge adequacy. CRMIs who had attended 6-10 normal deliveries (p = 0.039) and those who had witnessed more obstetric emergencies (p < 0.05) were more likely to have adequate knowledge compared to those with minimal exposure.

**Table 5:** Association of socio-demographic variables with knowledge about managing obstetric emergencies among study participants (n = 100) OBG: obstetrics and gynaecology Logistic regression. *p-value < 0.05 is statistically significant

Category	Inadequate knowledge (n = 16) n (%)	Adequate knowledge (n = 84) n (%)	OR (95% CI)	p-value
Gender				
Male	9 (19.6%)	37 (80.4%)	1.56 (0.58–4.22)	0.610
Female	7 (13.0%)	47 (87.0%)	Ref	
Currently posted in OBG				
No	8 (26.7%)	22 (73.3%)	1.84 (1.01–8.02)	0.047*
Yes	8 (11.4%)	62 (88.6%)	Ref	
Previously completed OBG posting				
No	5 (29.4%)	12 (70.6%)	1.74 (1.51-8.48)	0.043*
Yes	11 (13.3%)	72 (86.7%)	Ref	
Formal training in obstetric emergencies				
No	15 (18.3%)	67 (81.7%)	0.80 (0.46-0.92)	0.021*
Yes	1 (5.6%)	17 (94.4%)	Ref	
Number of normal deliveries attended				
0	4 (33.3%)	8 (66.7%)	0.78 (0.22-4.86)	0.312
1–5	4 (20.0%)	16 (80.0%)	1.13 (0.49-9.26)	0.971
6–10	3 (11.5%)	23 (88.5%)	2.68 (1.08-20.2)	0.039*
>10	5 (11.9%)	37 (88.1%)	Ref	-
Number of obstetric emergencies witnessed				
0	4 (30.8%)	9 (69.2%)	0.81 (0.25-6.52)	0.231*
1–5	4 (18.2%)	18 (81.8%)	2.52 (1.55-11.5)	0.041*
6–10	3 (8.6%)	32 (91.4%)	4.24 (1.12-24.3)	0.035*
>10	5 (8.9%)	51 (91.1%)	Ref	-

## Discussion

The present study demonstrated encouraging competency levels among CRMIs, with 84% showing adequate knowledge, 86% maintaining a positive attitude, and 74% demonstrating good practice in obstetric emergency management. These findings indicate that internship-level exposure, combined with structured training opportunities available during undergraduate rotations, has contributed to strengthening emergency preparedness among interns. When viewed alongside existing global evidence, the results reaffirm the value of competency-based and simulation-oriented education in improving both cognitive and practical readiness for maternal and neonatal emergencies.

International literature consistently highlights the benefits of structured emergency training in improving clinical performance. Niermeyer’s large-scale evaluation showed that the Helping Babies Breathe (HBB) programme significantly enhanced correct ventilation within the “Golden Minute,” reducing early neonatal mortality by up to 47% through repetitive simulation and standardized action plans [15]. In our study, CRMIs who underwent formal obstetric emergency training demonstrated significantly higher knowledge scores (p = 0.021), supporting the premise that structured simulation modules enhance retention of critical algorithms and improve psychomotor coordination. This parallel underscores the universal effectiveness of hands-on, algorithm-driven training across diverse learning environments.

Team-based obstetric emergency care, emphasized in global training models, also appears to shape intern attitudes in our setting. Bergh et al. reported substantial increases in teamwork scores following multiprofessional obstetric and neonatal emergency training in South Africa, with adherence to resuscitation protocols improving markedly [16]. Concordantly, 85% of CRMIs in our study valued simulation drills, and an equal proportion expressed confidence in inter-professional communication. These findings highlight that collaborative training environments elevate situational awareness, foster shared responsibility, and facilitate supportive hierarchy-qualities essential for ensuring safe and coordinated emergency responses.

Simulation-based training has repeatedly demonstrated its capacity to improve emergency response times and reduce clinical errors. Crofts et al. showed that targeted simulation training for shoulder dystocia significantly reduced decision-to-delivery intervals from 3.2 to 1.8 minutes [17]. Similarly, our CRMIs demonstrated strong foundational understanding of key emergency protocols, with 74% correctly identifying the McRoberts manoeuvre and 91% identifying oxytocin as the first-line uterotonic for PPH. Although our study assessed theoretical knowledge rather than real-time performance, the alignment suggests that simulation-based exposure contributes to faster recognition and more accurate decision-making during obstetric crises.

Beyond technical skills, sociological fidelity, replicating real-life team interactions and hierarchical dynamics, is increasingly recognized as essential for effective transfer of learning. Sharma et al. emphasized that introducing leadership pressure and interpersonal challenges into simulations enhanced psychological readiness and reduced anxiety in real emergencies [18]. In the present study, although most CRMIs exhibited high confidence, approximately 15% reported hesitation when unassisted. This finding suggests that while technical competence is increasing, psychological preparedness may lag slightly, reinforcing the need for simulations that include communication breakdowns, leadership stressors, and time pressure to strengthen emotional resilience among interns.

Another crucial factor influencing the effectiveness of emergency training is repetition. Siassakos et al. identified regular practice, feedback, and structured debriefing as major contributors to skill retention and error reduction during simulated obstetric haemorrhage scenarios [19]. This aligns with our observation that CRMIs who were currently posted in OBG, or had previous postings, demonstrated significantly higher knowledge adequacy (p = 0.047 and p = 0.043). Continuous exposure, whether through repeated postings, regular participation in drills, or frequent involvement in real emergencies, reinforces learning and deepens competence. The consistency between these findings and established global evidence underscores the importance of repetition and reflective feedback cycles within internship training.

Similarly, Draycott et al. demonstrated improved neonatal outcomes and reduced prevalence of low Apgar scores following routine shoulder dystocia training, emphasizing the direct link between simulation practice and clinical outcomes [20]. Although our study did not evaluate maternal or neonatal outcomes, the high practice adequacy rate (74%) and the substantial proportion of interns who had independently administered emergency drugs (76%) suggest that CRMIs are translating theoretical understanding into functional preparedness. This practice-performance synergy mirrors the patterns reported by Draycott et al. and highlights the potential for improved outcomes if such preparedness is consistently reinforced.

Effective PPH management, one of the leading causes of maternal mortality, also benefits significantly from simulation-based training. Nelissen et al. demonstrated an increase in correct uterotonic use from 42% to 93% following simulation interventions in Tanzania [21]. The present study similarly found strong theoretical understanding of PPH management, with 91% identifying oxytocin correctly. The alignment across studies suggests that even brief simulation exposure enhances rapid decision-making and escalation skills, which are critical for improving maternal survival in low-resource settings.

The educational advantages of simulation extend beyond obstetrics to multiple clinical disciplines. Deering et al. found that participants in structured obstetric simulation workshops achieved significantly higher post-test scores and exhibited increased confidence in managing high-risk scenarios such as cord prolapse and eclampsia [22]. Similarly, our CRMIs with repeated postings and higher exposure to emergencies achieved higher knowledge and attitude scores, reinforcing the benefit of progressive simulation-based learning from undergraduate to internship levels.

Improved teamwork and communication-central themes across emergency disciplines-also reflect in our findings. Andreatta et al. demonstrated that mock code simulations increased pediatric cardiac arrest survival by 25% due to better synchronization and task allocation [23]. In our study, 69% of interns regularly referred to emergency protocols, and 81% consistently escalated high-risk cases to seniors. These behaviours reflect the consolidation of critical-action pathways similar to those seen in pediatric simulation programs. Knight et al. also reported improved compliance with algorithmic steps and reduced medication errors following pediatric resuscitation team training [24], paralleling our observation of strengthened adherence to escalation protocols among CRMIs.

Simulation conducted directly within clinical settings (“in-situ simulation”) also provides substantial safety benefits. Patterson et al. identified previously unrecognized safety threats in 32% of emergency department simulations, reinforcing the importance of practising in real clinical environments [25]. Our observation that 70% of interns were posted in OBG during data collection and 81% had witnessed multiple emergencies supports the argument for immersive, context-specific learning opportunities.

Similar improvements in procedural success following simulation were reported by Kessler et al., who found that interns trained through simulation achieved a 91% success rate compared with 52% among untrained peers [26]. In our study, interns who had witnessed 6-10 emergencies demonstrated significantly higher knowledge adequacy (p = 0.035), strengthening the evidence that experiential learning frequency correlates strongly with technical proficiency.

Communication-focused simulation models, such as those described by Dadiz et al., have further demonstrated enhanced team dynamics during neonatal resuscitation [27]. This mirrors our findings, where 85% of CRMIs felt confident communicating during critical events and 80% reported confidence in administering emergency medications. Tools such as the CATS assessment validated by Frankel et al. have also shown improvements in teamwork scores and reductions in safety incidents [28], consistent with our interns’ strong attitudes toward structured training and feedback. Guise et al. similarly emphasized communication and situational awareness as predictors of improved maternal outcomes [29]; our observation that interns with greater exposure had better knowledge and practice levels aligns with these findings.

The study was limited by its single-centre design and a sample size restricted to 100 CRMIs, which may limit generalizability. Self-reported measures may have introduced recall or social desirability bias. Practical skills were not objectively assessed through direct observation or simulation performance metrics. Future multicentric and longitudinal studies incorporating pre- and post-training evaluations, objective skill assessments, and direct observation in simulated and clinical settings are warranted to validate these findings and measure the actual impact of structured obstetric emergency training on patient safety and clinical outcomes.

The findings highlight the need to institutionalize structured simulation-based obstetric emergency training within internship programs. Regular refresher drills, competency assessments, and integration of teamwork and communication modules can strengthen emergency preparedness further. Establishing dedicated skill-labs and in situ simulation frameworks could bridge existing gaps and promote long-term retention. Ultimately, improving CRMI preparedness has direct implications for reducing maternal morbidity and mortality, reinforcing the critical role of structured, experiential learning in enhancing obstetric emergency care.

## Conclusions

This study demonstrated that CRMIs at a tertiary care teaching hospital possessed encouraging levels of preparedness for obstetric emergency management, with 84% showing adequate knowledge, 86% demonstrating a positive attitude, and 74% exhibiting good practice. These findings indicate that internship exposure, combined with existing structured learning opportunities, has contributed to the development of essential competencies required for managing life-threatening obstetric conditions. Nonetheless, gaps remain among a subset of interns, particularly in areas related to consistent use of emergency protocols, confidence without supervision, and practical application of skills. The strong association between prior clinical exposure, structured training, and higher KAP scores highlights the importance of simulation-based education, hands-on drills, and repeated clinical immersion in strengthening emergency readiness. Enhancing these training components, along with regular refresher sessions, structured assessments, and interprofessional learning opportunities, could help bridge remaining gaps and ensure uniform competency across all interns. Overall, the study reinforces the need for institutionalized, competency-based obstetric emergency training for CRMIs as a strategy to improve maternal safety. Strengthening these training initiatives has the potential to translate into better clinical decision-making, timely interventions, and improved maternal and neonatal outcomes in tertiary healthcare settings.

## Appendices

**Table 6:** Structured KAP questionnaire WHO: World Health Organization; KAP: knowledge, attitude, and practices; OBG: obstetrics and gynaecology; ALSO: Advanced Life Support in Obstetrics; BLSO: Basic Life Support in Obstetrics; AMTSL: active management of the third stage of labour Questionnaire developed by Karuvizhi PM, TS Meena, and Reshmi S

Section/item	Question/statement	Response options
Section A: Demographic profile	Tool: WHO KAP survey model (adapted)	
A1	Age (in years)	__________
A2	Gender	◯ Male ◯ Female ◯ Others
A3	Internship batch/month of start	__________________
A4	Currently posted in OBG?	◯ Yes ◯ No
A5	Previously completed an OBG posting?	◯ Yes ◯ No
A6	Formal training in obstetric emergencies (ALSO/BLSO)?	◯ Yes ◯ No
A7	Number of normal deliveries attended	◯ 0 ◯ 1–5 ◯ 6–10 ◯ >10
A8	Number of obstetric emergencies witnessed	◯ 0 ◯ 1–5 ◯ 6–10 ◯ >10
Section B: Knowledge assessment	Sources: ALSO Curriculum, WHO MCPC, Laerdal Drills	
B1	First-line drug for postpartum hemorrhage	◯ Methylergometrine ◯ Oxytocin ◯ Misoprostol ◯Tranexamic Acid
B2	Therapeutic agent for eclampsia	◯ Diazepam ◯ Labetalol ◯ Magnesium sulfate ◯ Nifedipine
B3	A partograph is used for	◯ Monitoring fetal growth ◯ Estimating blood loss ◯ Monitoring labor progress ◯ Recording postpartum vitals
B4	Initial management of shoulder dystocia	◯ Uterine massage ◯ McRoberts maneuver ◯ Fundal pressure ◯ Emergency cesarean
B5	Red-flag sign for maternal sepsis	◯ BP 120/80 ◯ Temp 98.6°F ◯ Pulse >100/min ◯Urine output >50 mL/hr
B6	Recommended IV loading dose of MgSO₄	◯ 2 g IV ◯ 4 g IV ◯ 10 g IM ◯ 5 g IV + 5 g IM
B7	NOT part of AMTSL	◯ Controlled cord traction ◯ Uterine massage ◯ Prophylactic uterotonic ◯ Episiotomy
B8	“Golden hour” in obstetric referral	◯ 30 min ◯ 1 hr ◯ 2 hr ◯ 4 hr
B9	Most common cause of maternal death in India	◯ Sepsis ◯ Eclampsia ◯ Haemorrhage ◯ Obstructed labor
B10	Best assessment for uterine atony	◯ Fundal height ◯ Vaginal exam ◯ Palpating uterine tone ◯ Observing lochia
Section C: Attitude assessment	5-point Likert scale (1 = SD → 5 = SA)	Response: ◯1 ◯2 ◯3 ◯4 ◯5
C1	I feel confident managing postpartum hemorrhage.	1–5
C2	My UG training prepared me well for obstetric emergencies.	1–5
C3	I hesitate without supervision in emergencies.	1–5
C4	I am confident administering emergency drugs.	1–5
C5	Simulation-based drills should be mandatory.	1–5
C6	I fear making mistakes in emergencies.	1–5
C7	I feel confident using a partograph.	1–5
C8	I communicate well with team members in critical scenarios.	1–5
C9	I can take responsibility when required.	1–5
C10	Structured training will improve my performance.	1–5
Section D: Practice assessment	WHO/UNFPA EmOC Indicators (adapted)	
D1	Handled an obstetric emergency independently?	◯ Yes ◯ No
D2	Administered emergency obstetric drugs?	◯ Yes ◯ No
D3	Used a partograph during labor monitoring?	◯ Yes ◯ No
D4	Frequency of escalating high-risk labor to seniors	◯ Always ◯ Often ◯ Sometimes ◯ Rarely ◯Never
D5	Frequency of monitoring vitals in labor patients	◯ Always ◯ Often ◯ Sometimes ◯ Rarely ◯Never
D6	Participated in obstetric emergency simulation/drill	◯ Yes ◯ No
D7	Frequency of referring to emergency protocols	◯ Always ◯ Often ◯ Sometimes ◯ Rarely ◯Never
